# Conditional permutation importance revisited

**DOI:** 10.1186/s12859-020-03622-2

**Published:** 2020-07-14

**Authors:** Dries Debeer, Carolin Strobl

**Affiliations:** 1 University of Zurich, Psychological Methods, Evaluation and Statistics, Binzmuehlestrasse 14, Box 27, Zurich, 8050 Switzerland; 2 KU Leuven, Faculty of Psychology and Educational Sciences, Etienne Sabbelaan 51 box 7654, Kortrijk, 8500 Belgium; 3 KU Leuven, imec research group ITEC, Etienne Sabbelaan 51 box 7654, Kortrijk, 8500 Belgium

**Keywords:** Conditional permutation importance, Random forest, R

## Abstract

**Background:**

Random forest based variable importance measures have become popular tools for assessing the contributions of the predictor variables in a fitted random forest. In this article we reconsider a frequently used variable importance measure, the Conditional Permutation Importance (CPI). We argue and illustrate that the CPI corresponds to a more partial quantification of variable importance and suggest several improvements in its methodology and implementation that enhance its practical value. In addition, we introduce the threshold value in the CPI algorithm as a parameter that can make the CPI more partial or more marginal.

**Results:**

By means of extensive simulations, where the original version of the CPI is used as the reference, we examine the impact of the proposed methodological improvements. The simulation results show how the improved CPI methodology increases the interpretability and stability of the computations. In addition, the newly proposed implementation decreases the computation times drastically and is more widely applicable. The improved CPI algorithm is made freely available as an add-on package to the open-source software R.

**Conclusion:**

The proposed methodology and implementation of the CPI is computationally faster and leads to more stable results. It has a beneficial impact on practical research by making random forest analyses more interpretable.

## Background

Although they were originally developed for prediction purposes, Random Forests (RFs) [1] have become a popular tool for assessing the relevance of predictor variables in predicting an outcome^1^. Rather than applying a RF merely as a black-box prediction algorithm, so-called variable importance measures have been proposed and implemented to obtain an importance ranking of the predictors in fitted RFs, or to identify (or recursively select) a set of important predictors (i.e., variable selection). This article mainly focuses on identifying and ranking the predictors that play a role in achieving the prediction accuracy of a fitted RF, in the spirit of interpretable machine learning. However, the methods discussed below can in principle also be applied in variable selection algorithms.

Originally Breiman and Cutler [1, 2] proposed two variable importance measures: the Mean Decrease in Impurity (MDI) and — the focus of this article — the Mean Decrease in Accuracy, which we will refer to as the Permutation Importance (PI). During the last decade various alternative RF-based importance measures followed, resulting in a variety of possible measures [3–6]. Every proposed RF-based importance measure aims to quantify the contribution of the predictors in the RF, but different strategies are used. Generally a distinction can be made between importance measures that are based on the structure of the trees within a RF, such as the Intervention in Prediction Measure proposed by Epifanio [6] or the Minimal Depth measure by Ishwaran and colleagues [4], and measures that rely on the comparison of the prediction accuracy before and after noising up the predictor of interest, such as Breiman’s PI [1], or the importance measures proposed by Ishwaran [3] and Strobl et al. [5]. Many of the proposed measures have been empirically applied and have proven their practical value in a variety of different research fields. Some examples can be found in [7–11].

### Marginal vs. partial importance

Despite the practical value, and in contrast with their often clearly stated mathematical formulas and algorithms, it is generally unclear what the proposed measures exactly measure. That is, there is no consensus about what variable importance means theoretically, nor about how it should be operationalized [12]. Consequently, there is no general agreement on how a variable importance measure should ideally behave. This debate is not limited to RF-based importance measures. Even for the more basic case of linear regression, researchers hold different and opposing views on the interpretation and operationalization of variable importance. One example of this debate in linear regression can be found in the ongoing disagreement on the variable importance measure proposed by Hoffman [13]. It is advocated by some [14, 15], but strongly rejected by others [16–18]. Note that in the regression literature, variable importance is more commonly referred to as relative importance [12].

Summarized briefly, two extreme positions on variable importance can be discerned. First, there is marginal importance, which can be interpreted as the impact of a predictor for predicting the outcome without taking any other predictors into account. In linear regression this marginal importance corresponds to the (squared) zero-order correlation. Second, there is what we call partial importance, sometimes also referred to as conditional importance, which can be interpreted as the impact of a predictor on top of all the other predictors in the model. In linear regression the partial importance corresponds to, for instance, the (squared) semi-partial correlations. When all the predictors are independent, there is no difference between marginal and partial importance. However, in cases where there is some dependence structure between the predictors — in linear regression this implies that at least some predictors are correlated — the marginal and partial importance will differ. In these cases, marginal and partial importance can be seen as two extremes on one continuum. All the variable importance measures that have been proposed within the linear regression framework can be placed somewhere on this marginal-partial importance dimension (for an overview, see [12]), corresponding to a more partial, or more marginal perspective. Various authors [19, 20] have argued that any reasonable variable importance measure should incorporate parts of both the marginal and partial perspective, and hence, should correspond to some intermediate position on the marginal-partial importance dimension.

Because there is no consensus about what variable importance is or what it should be, it is impossible to identify the true or the ideal position for a variable importance measure on this dimension. Moreover, each researcher can subjectively decide which position on the dimension — and hence which proposed importance measure — best corresponds to his or her perspective on variable importance and to the current research question.

For a simplified example, consider the situation where a pharmaceutical company has developed two new screening instruments (Test A and Test B) for assessing the presence of an otherwise hard to detect disease. A study is set up, where the two screening instruments are used on the same persons. Due to time/money restrictions, only one screening instrument can be chosen for operational use. In this case, a more marginal perspective will be the preferred option to select either Test A or Test B. For instance, the test that has the strongest association with the presence of the disease (e.g., in the spirit of a zero-order correlation) can be chosen.

In contrast, let’s assume there already is an established screening instrument (Test X), and that the pharmaceutical company has developed two new screening instruments (Test A and Test B) of which only one can be used in combination with the established instrument Test X. In this case, a more partial perspective has our preference, as it assesses the existence and strength of a contribution of either Test A or Test B on top of the established Test X. For instance, the test that shows the highest partial contribution on top of the established Test X (e.g., in the spirit of a semi-partial correlation) can be chosen to use in combination with Test X.

For an alternative example, consider a screening study on genetic determinants of a disease. A variable importance measure in the spirit of the marginal perspective would give high importance values to all genes or single-nucleotide polymorphisms (SNPs) that are associated with the disease. Each of these genes or SNPs can be useful for predicting the outbreak of the disease in future patients. A variable importance measure in the spirit of the partial perspective, however, would give high importance values to the causal genes or SNPs but lower importance to genes or SNPs associated with the causal ones due to proximity. This differentiation can be useful to generate hypotheses on the biological genesis of the disease. Hence, the question whether the marginal or partial perspective is more appropriate depends on the research question.

Note that the lack of consensus and the multitude of importance measures does not imply that one should not use these measures. Rather the contrary, by applying multiple variable importance measures that differ in the extent to which they reflect marginal/partial importance, one can get a better understanding of the predictive relevance of the predictors^2^.

### Variable importance in random forests

Translating this view on partial and marginal importance to RFs is not straightforward because RFs are inherently different from linear regression models. Linear regression models assume a specific statistical model for the outcome with linear additive predictor effects and independently and identically normally distributed residuals. In contrast, a RF is an algorithmic ensemble method that does not impose any statistical model on the outcome. Predictor effects can be non-linear and highly interactive, which generally makes them impossible to disentangle or describe in a closed form.

In addition, due to the lack of a statistical population model, variable importance measures in RFs cannot be interpreted with respect to characteristics of the population or the true data generating mechanism. Rather, they should be seen as quantifications of the extent to which a predictor plays a role in obtaining the prediction accuracy. Thus, their scope is limited to the fitted RF^3^. In contrast, when the model is correctly specified, variable importance measures in linear regression can be interpreted as pertaining to the relevance of the predictors in the assumed data generating model in the population.

While it is clear what marginal and partial effects or contributions are in linear regression models, this is not the case for RFs. Nevertheless, the 2008 article of Strobl and colleagues [5] can be seen as an attempt to introduce the concepts of marginal and partial importance into the RF-based variable importance measures. More specifically, the authors argued that in some cases a more partial perspective may be more relevant than a marginal perspective, also when applying RFs. They argued that the original PI should not be interpreted as a partial importance measure, but rather as a more marginal importance measure. In addition, they introduced the Conditional Variable Importance — which we will refer to as the Conditional Permutation Importance (CPI) — as a tool for quantifying a more partial importance in RFs.

Since its proposal, the CPI, which was implemented in the party package for the statistical software R [23], has become a popular variable importance measure in RFs. It has been applied in numerous studies across different research fields, from marine ecology [9] over neurology [7] and geography [10] to linguistics [11]. The broad use of the CPI illustrates its relevance and shows that there is an interest in RF-based importance measures with a more partial perspective.

In this manuscript we reconsider the CPI. Although we support the rationale behind the CPI, we believe its implementation can be improved. Several studies [6, 24] have reported computational issues (i.e., long computing times and error messages in certain cases). Although we resolved these issues in an update of the party package in 2018, we argue that there are still other aspects of the party CPI implementation^4^ that can be further improved. We will propose a new CPI implementation that is faster and more stable. In addition, its application is not limited to RFs that were fit using the party package, but also includes CPI computation for RFs fit using the randomForest package [25]. For reasons of avoiding additional dependencies in the existing party implementation, this new implementation has been placed in a separate R-package named permimp (short for permutation importance).

The remainder of this manuscript is organized as follows. In the next section we first discuss the original PI [1, 2] and subsequently the CPI as introduced by Strobl and colleagues [5]. In the section thereafter we explain and illustrate some of the issues with the CPI in its current party implementation. Then we propose the new implementation, which we will refer to as the permimp implementation, as an attempt to mitigate these issues. Subsequently, we will focus on the threshold value in the CPI algorithm and introduce it as a parameter that can be modified to make the CPI less or more conditional, which, we argue, corresponds to moving the CPI along the marginal-partial importance dimension. Finally, using specifically simulated data, the impact of the threshold value is investigated and the performances of the permimp and the party implementation are compared.

### Original permutation importance (PI)

The original PI [1, 2] can be applied to the original RFs based on impurity reduction [1], to RFs based on the conditional inference framework [26], as well as to RFs grown using alternative algorithms [27, 28]. For a discussion of RF methods, see for instance [29] as well as the original publications. The rationale behind the PI is the following. When an outcome *Y* and a specific predictor *X*_*k*_ have some dependency structure, so called “noising-up”[3] *X*_*k*_ should destroy this dependency structure. Note that it is assumed that a relevant dependency between *X*_*k*_ and *Y* results in multiple splits with respect to *X*_*k*_ in the trees of the RF, thereby contributing to the prediction accuracy of the RF. However, when in a fitted RF the splits with respect to *X*_*k*_ are changed into random splits (i.e., noised-up), this contribution to the prediction accuracy will be lost. A random permutation of the values of *X*_*k*_ within a set of observations is one way to noise-up *X*_*k*_, i.e., to make the splits in a RF with respect to *X*_*k*_ random. Therefore, the difference in prediction accuracy of a RF before and after permuting the *X*_*k*_-values can be seen as a quantification of the importance of *X*_*k*_ in predicting the outcome *Y*. When there is practically no difference in the prediction accuracy before and after permuting *X*_*k*_, *X*_*k*_ is said to be unimportant. However, a lower prediction accuracy after permuting *X*_*k*_, and hence a positive difference indicates that the splits in the RF based on *X*_*k*_ were not just random, implying that *X*_*k*_ is important for predicting *Y*^5^.

The PI applies this rationale and computes the difference in prediction accuracy before and after permuting the values of *X*_*k*_. However, the permutation scheme is applied tree-wise, using only the out-of-bag (OOB) sample. The benefit of this is twofold. First, by using a different permutation for every tree and by averaging the prediction accuracy differences over the trees, the results become more reliable. Second, by using the OOB sample rather than the in-bag (IB) sample, the prediction accuracy before permuting is less likely to be overly optimistic. Thereby, positively biased PI-values for predictors that do not have any contribution are avoided. Note that recently OOB-based versions of the MDI have also been proposed [30, 31].

More formally, let *R*^(*t*)^ and $R^{(t)}_{(k)}$ be the prediction error of tree *t* in a RF with *p* predictors and ntree trees, based on OOB sample *β*^(*t*)^, respectively before and after permuting the OOB values of *X*_*k*_. For classification trees,

1 $$  \begin{aligned} R^{(t)} = & \sum_{i \in \beta^{(t)}} \frac{I \left(\hat{y}_{i}^{(t)} \neq y_{i}\right)}{\left|\beta^{(t)}\right|}, \\ R^{(t)}_{(k)} = & \sum_{i \in \beta^{(t)}} \frac{I \left(\hat{y}_{i(k)}^{(t)} \neq y_{i}\right)}{\left|\beta^{(t)}\right|}, \\ \end{aligned}  $$

and for regression trees,

2 $$  \begin{aligned} R^{(t)} = & \sum_{i \in \beta^{(t)}} \frac{\left(\hat{y}_{i}^{(t)} - y_{i}\right)^{2}}{\left|\beta^{(t)}\right|}, \\ R^{(t)}_{(k)} = & \sum_{i \in \beta^{(t)}} \frac{\left(\hat{y}_{i(k)}^{(t)} - y_{i}\right)^{2}}{\left|\beta^{(t)}\right|}. \\ \end{aligned}  $$

**Fig. 1:**
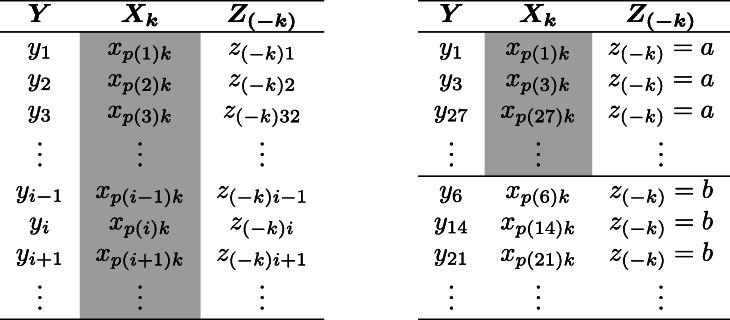
Permutation scheme for the original PI (left) and for the CPI (right). In the permutation scheme of the original PI (left) the values of *X*_*k*_ are permuted against both *Y* and *Z*_(−*k*)_. In the permutation scheme of the CPI (right) the values of *X*_*k*_ are permuted against *Y* conditionally on the values of *Z*_(−*k*)_

In Eqs. 1 and 2, $\hat {y}_{i}^{(t)} = f^{(t)}(\boldsymbol {x}_{i})$ is the RF prediction of OOB observation *i* before, and $\hat {y}_{i(k)}^{(t)} = f^{(t)}\left (\boldsymbol {x}_{i(k)}\right)$ with $\boldsymbol {x}_{i(k)} = \left (x_{i1}, \dots, x_{ik-1}, x_{p(i)k}, x_{ik+1}, \dots, x_{ip}\right)$, where *x*_*p*(*i*)*k*_ is the *i*’th observation of *X*_*k*_ after the permutation. *I*() is the indicator function and |*β*^(*t*)^| is the cardinal number of the OOB sample for tree *t*. The left panel of Fig. 1 presents the unconditional permutation scheme applied by the PI.

Both for classification and regression trees, the tree-wise permutation importance for *X*_*k*_ is:

3 $$  PI^{(t)}_{(k)} = R^{(t)}_{(k)} - R^{(t)}.  $$

The forest-wise permutation importance for *X*_*k*_ is the average over all tree-wise $PI^{(t)}_{(k)}$:

4 $$  PI_{(k)} = \frac{\sum_{t=1}^{\text{\texttt{ntree}}}PI^{(t)}_{(k)}}{\text{\texttt{ntree}}},  $$

where ntree is the number of trees in the RF.

Strobl and colleagues [5] related the PI to permutation tests [32], and argued that the hypothesis under which the permutation of one predictor *X*_*k*_ would not affect the prediction accuracy is the hypothesis of marginal independence between *X*_*k*_ and both the outcome *Y* and the other predictors $Z_{(-k)} = X_{1}, \dots, X_{k-1}, X_{k+1}, \dots, X_{p}$:

5 $$  X_{k} \perp Y, Z \: \: \: \text{ or} \: \: \: X_{k} \perp Y \wedge X_{k} \perp Z_{(-k)}.  $$

A PI value close to zero then corresponds to the marginal independence hypothesis. A large positive value, however, corresponds to a deviation from the hypothesis, which can be either a violation of the independence between *X*_*k*_ and *Y*, a violation of the independence between *X*_*k*_ and *Z*_(−*k*)_, or both. Simulation studies [24] have indeed shown that even when there is no dependence between the outcome and any of the predictors (*X*_*k*_⊥*Y* holds for all *X*_*k*_), highly correlated predictors (i.e., *X*_*k*_⊥*Z*_(−*k*)_ does not hold) have a positive PI.

Although often useful, researchers may not always be interested in this more marginal dependence (see, e.g., the examples presented above). Often the partial or conditional dependence between predictor and outcome is of interest. That is, the dependence between a predictor and the outcome conditionally upon the values of other predictors:

6 $$  (X_{k} \perp Y) | Z_{(-k)}.  $$

Therefore, Strobl and colleagues [5] proposed a permutation importance measure that applies a conditional permutation scheme, namely the CPI.

### Conditional permutation importance (CPI)

The CPI can also be formulated using Eqs. 1 to 4, with the difference that for each tree the OOB values of *X*_*k*_ are permuted conditionally on the values of *Z*_(−*k*)_. To be more precise, the predictor space is partitioned based on *Z*_(−*k*)_ and within each partition *Z*_(−*k*)_=*z*_(−*k*)_ the OOB values of *X*_*k*_ are conditionally permuted. Figure 1 illustrates the difference between the original and the conditional permutation scheme. In the left panel of Fig. 1 (cf. PI) the values of *X*_*k*_ are permuted unconditionally. In contrast, in the right panel (cf. CPI), the values of *X*_*k*_ are only permuted within groups of observations for which *Z*_(−*k*)_=*z*_(−*k*)_.

Deciding a reasonable and computationally feasible partitioning for the conditional permutation is not straightforward. Therefore, Strobl and colleagues [5] proposed to define the partitions for the conditional permutation based on the predictor-space partitioning induced by the tree-growing algorithm. The main advantage of this approach is that the tree-growing-based partitioning (a) is already learned from the data and, hence, easily accessible; (b) does not depend on the OOB values, and thereby avoids creating the permutation scheme and computing the prediction accuracy using the same observations; and (c) also contains clear splits in non-categorical predictors. Strobl and colleagues proposed the following two-step strategy, which was implemented as a function in the party R-package^6^.

#### Step 1.

In the first step, for each predictor *X*_*k*_ it is determined which other predictors are included in *Z*_(−*k*)_, which is the set of predictors to base the conditional permutation on. Rather than including all the predictors (except *X*_*k*_) in *Z*_(−*k*)_ — which would be over cautious, severely restrict the size of the partitions, and, therefore, limit the impact of the permutation — only those predictors that are, to a certain extent, related to *X*_*k*_ are included. To be more precise, the association between *X*_*k*_ and the other predictors in *Z*_(−*k*)_ is tested by applying the conditional inference framework of Hothorn and colleagues [33] to the complete data set^7^. Only those predictors for which the association with *X*_*k*_ is strong enough, i.e., for which the *p*-value of the used statistical test is small enough, are selected. That is, when 1− the *p*-value for the association test between *X*_*k*_ and another predictor *X*_*l*_ is bigger than a user-defined threshold value *s* (0≤*s*≤1), *X*_*l*_ is selected as a predictor to condition on. When (1−*p*)<*s*, *X*_*l*_ is not selected. The result is a subset of *Z*_(−*k*)_, which we refer to as $Z_{(-k)}^{(s)}$.

#### Step 2.

**Fig. 2:**
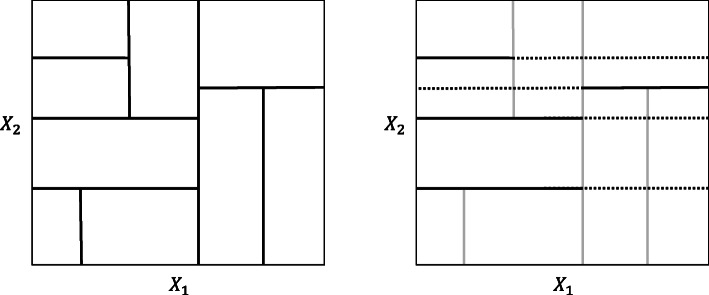
Tree-growing (left) and conditional permutation (CPI; right) predictor space partitioning. In the tree-growing partitioning (left), only one split completely splits the predictor space, all the other splits are conditional on the previous split(s). In the partitioning that is used for permutation scheme in the CPI, where the predictor of interest is *X*_*k*_=*X*_1_ and $Z_{(-k)}^{(s)} = X_{2}$ (right), the splits with respect to *X*_2_ are extended to completely split the predictor space (dashed lines) while the splits with respect to *X*_1_ are ignored (gray lines)

Once the set of predictors to condition on $Z_{(-k)}^{(s)}$ is selected, a second step is repeated for every tree *t* individually. All the split points in the tree *t* for the predictors in $Z_{(-k)}^{(s)}$ are combined to create a (multi-dimensional) grid that partitions the predictor space. It is important to note that in this grid each split completely bisects the predictor space. This is in contrast with the original tree-growing partitioning, where a split is always based on the previous split(s) so that most splits only bisect a limited subspace of the predictor space. Figure 2 illustrates the difference between the original tree-growing partitioning (left panel) and the partitioning that is used for permutation scheme in the CPI where the predictor of interest is *X*_*k*_=*X*_1_ and $Z_{(-k)}^{(s)} = X_{2}$ (right panel). The splits pertaining to *X*_2_ in the tree-growing partitioning (left panel) are extended for the CPI partitioning (right panel; dashed lines) so that they completely bisect the predictor space.

Although $Z_{(-k)}^{(s)}$ (decided using the complete data in the first step) is the same for every tree, the determined permutation schemes differ across trees. Because each tree is grown on different IB samples, it can be assumed that each tree has different splitting variables and split points. In addition, the OOB-values also differ across trees, thereby also determining the range of possible permutations.

In summary, for a predictor *X*_*k*_, the implementation of the CPI in the party package can be described as follows:

- (*Step 1*) Using the complete data:
  1. Choose a threshold value *s* (the default in the party package is *s*=.20).
  2. Select the predictors to condition on $Z_{(-k)}^{(s)}$, by applying the conditional inference framework, and including those predictors for which 1 - the *p*-value of the used test is bigger than *s*.
- (*Step 2*) Repeat for each tree *t* in the RF:
  1. Collect all the split points for the predictors in $Z_{(-k)}^{(s)}$.
  2. Create a partitioning grid by completely bisecting the predictor space using the collected split points.
  3. Compute the OOB prediction error *R*^(*t*)^.
  4. Within each partition of the partitioning grid, permute the OOB values of *X*_*k*_.
  5. Recompute the OOB prediction error $R^{(t)}_{(k)}$.
  6. Compute the tree-wise $CPI^{(t)}_{(k)}$.
- Average the tree-wise $CPI^{(t)}_{(k)}$ across all the trees to obtain *CPI*_(*k*)_

Simulation studies [5, 24] have shown that, in comparison with the original PI, the CPI can indeed be interpreted as a measure that determines a more partial impact on the prediction accuracy. In addition, when the outcome is independent from the predictors, the CPI does not show the preference for correlated predictors that has been observed in the PI [24].

In addition, Strobl et al. and others [5, 34, 35] have indicated that the mtry hyperparameter in the RF-algorithm affects the pattern of variable importance measures. However, because the scope of RF-based variable importance measures is limited to quantifying predictor contributions in the fitted RF, we argue that one should use that mtry-value — and by extension, those hyperparameter values in general — that optimize the prediction accuracy. Finding those optimal hyperparameter values can, for instance, be done using cross-validation. When the RF with the best prediction accuracy is found, variable importance measures can be applied to quantify the predictor contributions in obtaining these optimal predictions. In practice one should also check whether the prediction accuracy of the RF is satisfactory, or at least better than chance, because quantifying the contribution of a predictor in a bad prediction machine is useless^8^.

### Four issues

Due to its wide use in applied research, several issues related to the party implementation of the CPI have been discovered. In the following we discuss four issues, all of which we attempt to mitigate in the permimp implementation. The four issues pertain to (a) the computation speed, (b) a restriction to linear dependencies, (c) the sample-size dependency of the selection criterion, and (d) the instability of the CPI across simulated data. We will already compare the party and the new permimp implementation of the CPI with respect to these issues, before explaining the specifics of the new CPI implementation in the subsequent section: “permimp CPI Implementation”.

#### Computational speed

Several authors have reported the CPI implementation in the party package to be slow for larger sample sizes. Moreover, in some cases the algorithm failed due to its high storage needs [6, 24]. In a recent update of the party-package, we have already resolved the memory problems in the CPI algorithm, which also made the computation faster for bigger data sets. Nevertheless, we believe that an even faster computation would be beneficial for practical use, especially when dealing with larger sample sizes and a high number of predictors.

After inspecting the current CPI party implementation, we found several instances that could be further optimized. For instance, it is possible that, within the partitioning grid, there are partitions for which all observations end up in the same endnode. Permuting the *X*_*k*_-values of observations that fall within such a partition cannot affect the prediction accuracy, since the observations simply cannot end up in different end-nodes. One way to reduce the computing time of the CPI is to omit these redundant permutations. Further reorganizing and recoding the CPI algorithm in the permimp implementation resulted in a significant gain in speed compared to the current party implementation.

**Fig. 3:**
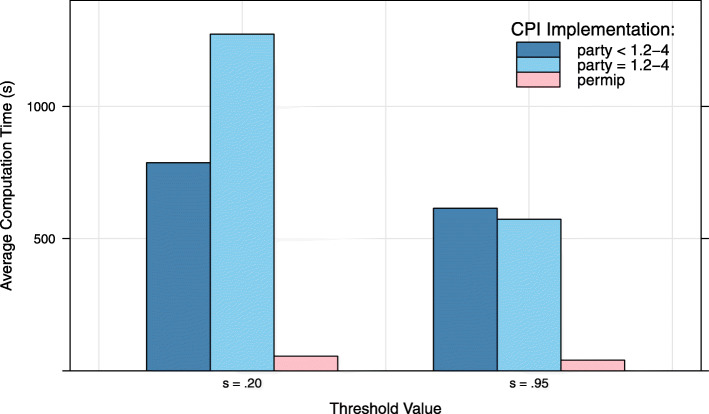
Computation speed: party vs. permimp implementation. Fifteen RFs (using ntree =1000 and mtry =10) were fit to a peptide-binding data set [5, 36]. The data set includes 310 observations of 105 predictors for a binary outcome. CPI according the old and the current party implementation as well as the permimp implementation were computed for two threshold values: *s*=.20 and *s*=.95. The average computation speed for the three implementations are given as respectively blue and red bars

To illustrate the gain in computation speed, the old party implementation (i.e., version <1.2-4), the current party implementation, and the permimp implementation of the CPI were applied to the peptide-binding data from the empirical example in [5]. The data set includes 105 variables for a total of *n*=310 amino acid sequences. The outcome to be predicted is a binding property that can be coded as a binary variable (binding/no binding). More information about this data set can be found in [5] and [36]. Fifteen RFs with ntree =1000 trees and mtry =10 were fit to the data and the CPI in the three implementations was computed for each RF. Figure 3 presents the average speed across the 15 RFs for the three implementations, using threshold values *s*=.2 (the default in party) and *s*=.95 (the default in permimp). Although similar CPI values were obtained across all implementations for both threshold values, the permimp implementation was on average more than ten times faster than the old and the current party implementation.

#### Linear association limitation

As described above, rather than conditioning on all the other predictors *Z*_(−*k*)_, only the predictors *X*_*l*_ that are associated with *X*_*k*_ are considered for the conditional permutation scheme of *X*_*k*_, leading to the set $Z_{(-k)}^{(s)}$. To select the predictors to condition on in $Z_{(-k)}^{(s)}$, the party implementation uses the conditional inference framework [33] to test the statistical independence between *X*_*k*_ and every *X*_*l*_. However, some of the tests within the conditional inference framework, such as its independence test for two continuous variables, are only sensitive to linear associations. As a result, for two continuous predictors *X*_*l*_ and *X*_*k*_, the party implementation of the CPI will not select *X*_*l*_ in $Z_{(-k)}^{(s)}$, even when there is, for example, a strong U-shaped dependence^9^ between *X*_*l*_ and *X*_*k*_.

Violations of statistical independence are of course not limited to linear associations, there can also be a non-linear dependency between two predictors. Given the inherent non-linear nature of RFs, we argue that it would be an advantage if the procedure that selects the *X*_*l*_ to be in $Z_{(-k)}^{(s)}$ is sensitive to both linear and non-linear associations. As will be explained below, the new permimp CPI implementation uses a different strategy for testing the independence between predictors, which was specifically chosen to be sensitive to both linear and non-linear associations.

**Fig. 4:**
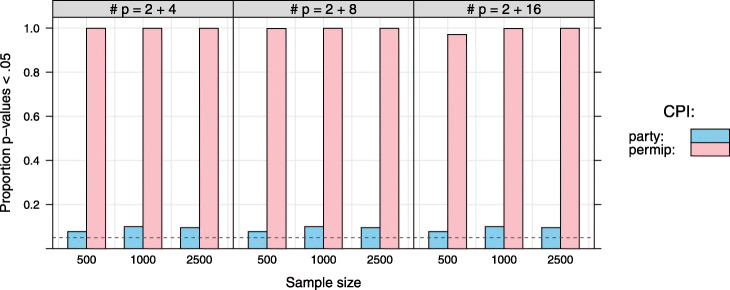
Non-linear dependencies: party vs. permimp implementation. Data sets were sampled with five, nine or seventeen uniformly distributed (min = -3, max = 3) predictors. An additional predictor was created by squaring one of the uniform predictors, so that all but two predictors were independent, and two showed a perfect quadratic relation. This resulted in two quadratically related predictors plus either four, eight or sixteen independent predictors. Sample size was either *N*=500,1000, or 2500, and either no, half or all the predictors had a linear impact on the continuous outcome variable. The dependence tests within the party and the permimp implementation (i.e., a *χ*^2^-test based on the tree-growing split points) were applied only to the two predictors with the perfect quadratic relation. The proportion of *p*-values lower than.05 within the party and permimp implementation are indicated in blue and pink respectively. The dashed line corresponds to a proportion of.05

Figure 4 compares the proportion of *p*-values below.05 according to the independence tests applied in the party and the permimp implementation to select predictors to condition on, for two predictors that are perfectly quadratically related. The party implementation, which relies on linear associations is not sensitive to the quadratic association between the predictors. In comparison, the permimp implementation consistently results in very small *p*-values in almost all cases^10^.

#### Sample size dependence

A second issue related to the selection of the predictors to condition on $\left (Z_{(-k)}^{(s)}\right)$ pertains to the sample size dependence of the applied methodology. Ideally, the predictors in $Z_{(-k)}^{(s)}$ should be selected based on the strength of their association with *X*_*k*_. However, the predictors in a RF can be of different variable types (i.e., categorical, ordinal or continuous), and there is no universal measure for association strength that is applicable to all pairs of *X*_*k*_ and *X*_*l*_, regardless of their variable types. Hence, combinations of different variable types require different association measures (e.g., correlation, rank-order correlation). Because these measures do not necessarily share the same scale, the effect sizes are not directly comparable. For instance, an effect size for the association between two categorical variables cannot be compared easily with an effect size for the association between two continuous variables. To overcome this issue, the party CPI implementation uses *p*-values, rather than raw effect sizes to decide whether or not to include a predictor *X*_*l*_ in $Z_{(-k)}^{(s)}$. Regardless of the combination of variable types, the *p*-values are always on the same scale.

There are, however, two downsides to this strategy. First, within a tree, only the location of the observations with respect to the split-points plays a role. That is, not the raw observation, but rather the tree-induced partition in which the observation falls is important. The tests to select the predictors to condition on, however, are based on the raw observations. In addition, each tree relies only on the IB data, while the applied tests use the complete data. Note that a dependency between the raw values in the complete data does not necessarily lead to a dependency in the tree-based partitioning based on the IB observations. As a result, despite a dependency between the raw values of *X*_*k*_ and *X*_*l*_, including *X*_*l*_ in $Z_{(-k)}^{(s)}$ could be redundant and inefficient when computing the CPI.

Second, *p*-values do not depend on the effect size solely, but also on the sample size. Although this is a valuable feature when applying significance testing in general — as well as when selecting the next split in the binary tree building algorithm [26] — it is inconvenient when the purpose is to select the other predictors $Z_{(-k)}^{(s)}$ to condition the permutation of the *X*_*k*_ values on. As a consequence, higher sample sizes lead to a more greedy selection of the predictors to condition on.

**Fig. 5:**
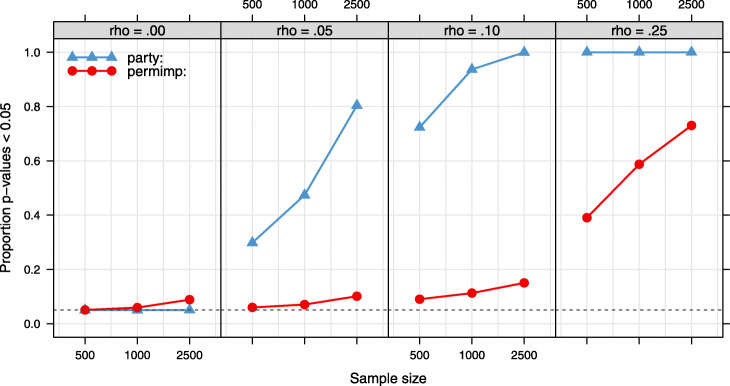
Sample size dependence: party vs. permimp implementation. Data sets were sampled with pairwise correlations of either *ρ*=.00,.05,.10, or.25 between 18 normally distributed predictors. Sample size was either *N*=500,1000, or 2500, and either no, half or all the predictors had a linear impact on the continuous outcome variable. The dependence tests within the party (correlation *t*-test) and the permimp implementation (*χ*^2^-test based on the tree-growing split points) were applied. The proportion of *p*-values lower than.05 within the party and permimp implementation are indicated in blue and pink respectively. The dashed line corresponds to a proportion of.05

Figure 5 illustrates that, when using the party implementation, the proportion of *p*-values lower than.05 rapidly increases with sample size, even when the effect size of the linear dependence is very small. As described above, only those predictors for which 1 minus the *p*-value of the independence test with *X*_*k*_ is higher than the threshold *s* are included in $Z_{(-k)}^{(s)}$. Consequently, the probability that a predictor is selected as a predictor to condition on increases rapidly with sample size, despite that predictor being only slightly associated to *X*_*k*_. In comparison, the permimp implementation seems to be less sensitive to the sample-size effect.

#### Instability

**Fig. 6:**
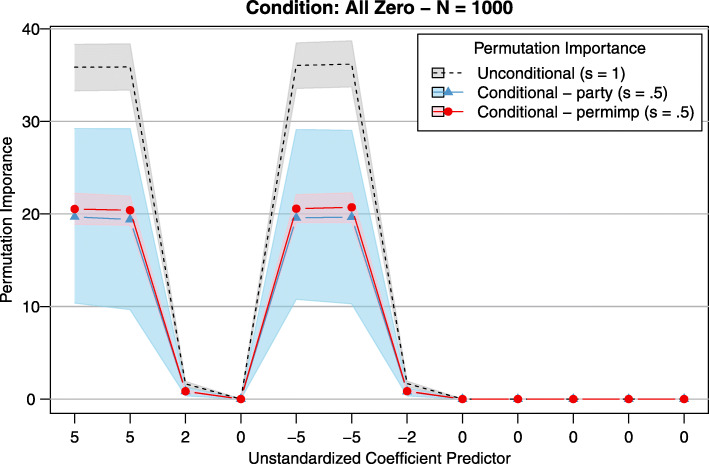
Stability of the CPI: party vs. permimp implementation. Data were generated according to a linear model with 12 uncorrelated normally distributed predictors, the regression coefficients were 5, 5, 2, 0, -5, -5, -2, 0, 0, 0, 0, and 0. In each of the 1000 replications 1000 observations were sampled, and a regression RF with 1000 trees was fit, after which the CPI was computed with a threshold value of *s*=0.5. The mean CPI (lines) as well as the region between the first and third quartile (shaded area) of the CPI distribution across the 1000 replications are depicted for every predictor, both for the party and the permimp implementation of the CPI, in blue and red, respectively. As a reference, the unconditional PI, which corresponds to the CPI with a threshold value of *s*=1 regardless of the implementation, is depicted in black. Because all predictors are independent, the PI and the CPI have demonstrate the same pattern

When repeatedly computing the CPI using simulated data, we noticed that the CPIs computed according to the party implementation can be unstable. In general, when sampling multiple data sets according to the same data generating mechanism it is normal that the sampling process causes some variation in the results. However, the instability demonstrated by the party CPI implementation seems to go beyond this sampling variation. As an illustration, Fig. 6 presents the distribution of the CPI of 12 continuous predictors across 1000 replications. Data were generated according to a linear model with the same regression coefficients as in the study of Strobl and colleagues [5]. All predictors were independent (cf. the *ρ*=0 condition in part 1 of the simulation study in the “Methods” section). In each replication, 1000 observations were sampled, and a regression RF with 1000 trees was fit to every generated data set, after which the CPI was computed with a threshold value of *s*=.5, along with the original PI, which corresponds to a CPI with a threshold value of *s*=1. Figure 6 displays both the mean CPI for every predictor, as well as the region between the first and third quartile of the CPI distribution across the 1000 replications. For the predictors with non-zero regression coefficients, the variability in CPI values (according to the party implementation) was clearly higher than the variability observed in the CPI values according to the permimp implementation and also higher than the variability observed in the unconditional PI values. Both CPI implementations, however, lead to about the same mean values, but the permimp implementation demonstrated better stability.

Further analyses indicated that this difference in stability between the two implementations is not limited to the raw CPI values, but is also observed when the order of the CPI values are considered, a common practice when working with variable importance measures [6, 29]. We believe that the instability of the party implementation can be explained (at least in part) by the way the predictors to condition on are selected. First, because the selection procedure is based on the whole data, it is the same for all trees in the forest. Second, for Fig. 6 the used threshold value was *s*=.5, which implies that predictors that are actually independent were nevertheless selected to condition on in about 50 percent of the replications. Therefore, across the replications, it is likely that there were both RFs for which multiple predictors were selected to condition on, as well as forests for which no predictors were selected to condition on. In other words, the number of predictors to condition is likely to differ strongly across the replications. Consequently, the differences between the CPI-values across replications were considerable.

### permimp CPI implementation

In this section we present the new permimp CPI implementation, which can be seen as an attempt to mitigate the above-raised issues pertaining to the party implementation. Besides more efficient coding, the permimp implementation differs from the party implementation in two main respects. Both differences pertain to the first step in the CPI algorithm, which selects the predictors to condition on $Z_{(-k)}^{(s)}$.

First, rather than using the complete data, we propose to select the predictors to condition on $Z_{(-k)}^{(s)}$ for every tree independently: $Z_{(-k)}^{(t)(s)}$. To be more precise, for every tree *t* the selection procedure is repeated using only the IB data for that tree. This strategy is more in line with the rationale behind RF as an algorithmic ensemble method, where a specific algorithm is executed repeatedly on bootstrapped or sub-sampled data, after which the results are aggregated.

In addition, the tree-wise strategy can protect against the instability of the party implementation observed in simulated data, because the randomness (and inherent instability) in the selection of the predictors to condition on is transferred from the forest-level to the tree-level. By combining a large number of trees in one forest, the permimp implementation averages out this inevitable randomness, and more stable results across replications are observed (cf. Fig. 6). Note that, based on our simulation study results, we also propose a more strict default threshold-value (*s*=.95).

The second difference between the permimp implementation and party implementation of the CPI relates to the values that are used to decide whether or not to select a predictor *X*_*l*_ in $Z_{(-k)}^{(t)(s)}$. Rather than using the raw observed predictor values, we propose to use discretized versions of the predictors. To be more precise, we propose to split every predictor *X*_*k*_ using its split-points in tree *t*, thereby creating discretized versions of the predictors: $X^{(d)}_{k}$, $X^{(d)}_{l}$ etc. For instance, a continuous predictor with two split points in tree *t*, would result in a categorical predictor with three levels. As a consequence, the *χ*^2^ independence test can be applied to all predictor combinations, rather than applying different tests depending on the variable types, such as in the conditional inference framework [33].

The benefit of this strategy is twofold. First, *χ*^2^-tests are sensitive to any violation of independence between categorical variables, and are therefore not restricted to linear associations (cf. Fig. 4). Second, this strategy seems to reduce — but not entirely solve — the sample-size dependence (cf. Fig. 5), thereby ensuring that the association strength is a more decisive factor when selecting the predictors to condition on.

One reason why one could object against the proposed implementation is related to the discretization. Because it leads to a reduction of the available information, discretizing non-categorical predictors is generally considered a bad practice [37]. We argue, however, that discretizing predictors is an inherent part of the decision tree methodology. Once a tree has been established, the raw predictor values are redundant, only whether the raw value is smaller or bigger than the selected split points is relevant. Hence, when discretizing a predictor according to its split points in the tree, all the information that is relevant within that tree is maintained.

For a predictor *X*_*k*_, the permimp implementation of the CPI can be described as follows:

- (*Step 1*) Repeat for each tree *t* in the RF:
  1. Choose a threshold value *s* (the default in the permimp package is *s*=.95).
  2. Discretize all the predictors with split points in tree *t* according to the split points in tree *t*.
  3. Select the predictors to condition on $Z_{(-k)}^{(t)(s)}$, by applying *χ*^2^-tests using the discretized IB values of tree *t*, and including those predictors for which 1 - the *p*-value of the *χ*^2^-test is bigger than *s*.
- (*Step 2*) Repeat for each tree *t* in the RF:
  1. Create a partitioning grid by completely bisecting the predictor space using the discretized version of the predictors in $Z_{(-k)}^{(t)(s)}$.
  2. Compute the OOB prediction error *R*^(*t*)^.
  3. Within each partition of the partitioning grid, permute the OOB values of *X*_*k*_.
  4. Recompute the OOB prediction error $R^{(t)}_{(k)}$.
  5. Compute the tree-wise $CPI^{(t)}_{(k)}$.
- Average the tree-wise $CPI^{(t)}_{(k)}$ across all the trees to obtain *CPI*_(*k*)_

#### Additional technical remarks

In the permimp implementation, regardless of the variable type, only one testing procedure is applied to select the predictors to condition on $Z_{(-k)}^{(t)(s)}$. Nevertheless, it is still impossible to compare the effect sizes (i.e., *χ*^2^-values) across the predictors. To be more precise, in a tree the number of split points differs across the predictors, implying that the discretized versions of the predictors will have different numbers of categories, which makes the raw *χ*^2^-values incomparable. Therefore, like the party implementation, the permimp implementation also relies on the associated *p*-values to include a predictor *X*_*l*_ in $Z_{(-k)}^{(t)(s)}$.

Note that in the permimp implementation, for each predictor *X*_*k*_ the set of predictors to condition on is made for every tree separately: $Z_{(-k)}^{(t)(s)}$, while in the party CPI implementation the selection is only made once for all trees: $Z_{(-k)}^{(s)}$. Of course, repeating this selection for every tree increases the computational burden. Yet by applying more efficient coding, the permimp implementation is generally faster than the party implementation, especially when the number of predictors grows (cf. Fig. 3).

Finally, the CPI as implemented in the permimp-package can be applied to RFs grown using the conditional inference framework (through the R-package party) [26], as well as to RFs that are grown according to the impurity reduction principle (through the randomForest package) [25]^11^. The party CPI implementation, in contrast, is limited to RFs fit using the party-package.

### Interpreting the threshold value: from marginal to partial

In this section, we argue that the threshold value in the permimp implementation can, to some extent, be viewed as a parameter that determines the position of the CPI on the marginal-partial dimension (cf. above)^12^. Because the CPI quantifies the impact of the predictors conditionally on the relevant other predictors in the RF, we assume it corresponds with a more partial perspective on variable importance. However, as explained above, the conditional permutation scheme depends on the chosen threshold value *s*. A lower threshold value increases the number of predictors *X*_*l*_ that are included in $Z_{(-k)}^{(t)(s)}$, thereby making the permutation scheme — and the CPI — more conditional. Therefore, following the rationale that the CPI can be seen as a more partial RF-based importance measure, we argue that the threshold value can be interpreted as a parameter that determines how partial the CPI is.

If the threshold indeed determines the position of the CPI on the marginal - partial continuum, we can expect the following behavior. First, when all predictors are independent, the chosen threshold should not affect the results. Likewise, in regression marginal and partial importance measures have similar results when all predictors are uncorrelated [12]. Second, when there is some dependency structure between the predictors, CPI patterns should differ for different threshold values, which would correspond to the observed differences between more partial and more marginal importance measures in linear regression with correlated predictors [12]. Finally, and ideally, the transition from more marginal to more partial CPI patterns should be monotone and somewhat smooth.

Before using simulated data to investigate whether and how the threshold value affects the CPI patterns, the following considerations about the impact of the threshold value on the CPI pattern can already be derived from the algorithm. First, when the threshold *s*=1, no predictors to condition on are included in $Z_{(-k)}^{(t)(s)}$, which corresponds to an unconditional permutation scheme. Therefore, the original PI can be seen as a special case of the CPI.

Second, when *s*=0, all other predictors *X*_*l*_ are included in $Z_{(-k)}^{(t)(s)}$, so that $Z_{(-k)}^{(t)(s)} = Z_{(-k)}^{(t)}$, making the permutation scheme as conditional as possible. Although it may seem appealing to interpret this most conditional case as corresponding with the CPI that is as partial as possible, there is an important caveat. The most conditional permutation scheme may lead to meaningless CPI values. Because all split-points from all predictors in the tree are used, the permutation scheme can become very elaborate and fragmented, with a high number of small partitions. Because in the permutation scheme all splits completely bisect the predictor space (cf. Fig. 2), the number of partitions can be larger than in the tree-based partitioning. In overly fragmented permutation schemes, each OOB observation can only be permuted with a limited set of other observations — if at all — thereby reducing the potential prediction accuracy reduction caused by permuting the predictor values. Consequently, all CPI values will be arbitrarily close to zero, and hence meaningless for quantifying the predictor contributions in the prediction.

Moreover, threshold values *s*≤0.5 imply that even when two predictors *X*_*k*_ and *X*_*l*_ are independent, each has a probability of ≥.5 of being selected in $Z_{(-l)}^{(s)}$ and $Z_{(-k)}^{(s)}$, respectively. Because conditioning on independent predictors will not have any meaningful impact on the CPI patterns, we expect that these threshold values will have limited practical relevance. Only the predictors that are associated with the predictor of interest should be selected to condition on. In other words, for *s*≤0.5 the selection of predictors to condition on may be too greedy. Consequently and in hindsight, the default threshold value of *s*=.2 in the party implementation can be considered a too liberal choice.

A final consideration about the impact of the threshold value pertains to the sample size. Because the selection of the predictors to condition on is based on a testing approach (i.e., *p*-values), unavoidably there is some sample size dependence. For instance, even when both the effect size of the association between two predictors and the applied threshold value are kept constant, increasing the sample size will increase the probability of including predictor *X*_*l*_ in $Z_{(-k)}^{(t)(s)}$, and may thus lead to different CPI patterns. This implies that the impact of the threshold value on the CPI pattern will depend on the sample size. Therefore, when choosing the threshold-value, the sample size should be taken into consideration: the bigger the sample size, the faster high threshold values may lead to a more greedy selection of the predictors to condition and thereby a more partial importance quantification.

## Results

A simulation study was set up to (a) investigate and illustrate the impact of the threshold *s* on the CPI values, and (b) compare CPI-results according to the party with the permimp implementation. The complete description of the design and the result can be found in the Methods section below. In addition, all results can be graphically browsed through using a shiny app^13^.

Briefly summarized, the results of the simulation study supported our claim that the threshold value can be interpreted as a parameter that makes the CPI more partial or more marginal. In the cases where all predictors were independent — and where the marginal and partial perspectives should lead to similar importance rankings — the PI and the CPI demonstrated similar patterns. That is, decreasing the threshold decreased the raw CPI values, but did not change the pattern of (C)PI values. In contrast, when there were dependencies between the predictors, decreasing the threshold did change the CPI pattern. In addition, in these cases — and in contrast to the PI patterns — the CPI patterns corresponded to what one would expect from a more partial importance measure.

The most important differences in the relative CPI pattern were observed for threshold values close to one. Decreasing the threshold value after *s*=.80 only reduced the raw CPI values, without meaningful changes in the CPI pattern. That is, the raw CPI values were decreasing (because more fragmented permutation schemes reduced the possible impact of permuting), but the relative CPI pattern stayed unchanged. There was, however, no evidence for too fragmented permutation schemes when *s*>0, and only limited evidence when *s*=0.

In addition, the results confirmed that the permimp implementation of the CPI is more stable than the party implementation, and that it is also sensitive to non-linear dependencies between predictors. Overall, the simulation study results indicate that the permimp implementation mitigates the above described issues pertaining to the party implementation of the CPI.

## Discussion

In this section, first practical recommendations related to the CPI and its threshold value are discussed. Subsequently, we critically discuss the limitations of our study and provide suggestions for future extensions.

### Practical recommendations

Often, after fitting a RF, researchers or practitioners want explore their “black-box” prediction machine. In many cases a more partial perspective on variable importance will be of interest, like for instance in the studies of [7, 9–11]. The CPI provides this perspective. Yet which threshold value should be used? We hold that there is not one optimal threshold value that corresponds to the “true” CPI. Indeed, the results from the simulation study show that the impact of the threshold value depends on the data (i.e., sample size, data generating mechanism, etc.). Therefore, when feasible, we recommend computing the CPI multiple times, using different threshold values (including the CPI with *s*=1, since this correspond to the unconditional PI). This will make it possible to detect changes in the CPI pattern, and discern between the importance of a predictor according to a more partial and a more marginal perspective. In addition, we believe that the changes in the CPI pattern when going from less to more conditional may be more informative than one single CPI pattern.

Based on the results of the simulation study, we chose a threshold value of *s*=0.95 as the default in the permimp package. We would advise against using a threshold value smaller than *s*=0.8, because these threshold values do not change the pattern of the CPI any more but only decrease the raw CPI values. Finally, users should be aware of the sample size dependence and consider using higher threshold values (i.e., closer to one) when dealing with larger sample sizes (cf. above).

### Limitations and extensions

Like the party implementation, the permimp implementation of the CPI can only be applied to data sets without missing data. To deal with missing data, many RF algorithms apply surrogate splits while growing the trees and while predicting the outcome [26]. However, these surrogate splits are problematic when defining the grid for the conditional permutation scheme. Hapfelmeier and colleagues [39] proposed an alternative to the PI that, by relying on the splitting proportions, does not require surrogate splits. A similar approach for the CPI, however, seems infeasible because it would require the computation of the conditional split proportions for every partition in the conditional “permutation” scheme, and this for every tree, which would increase the computational cost exponentially. From a practical perspective, we would propose to use (multiple) imputations to deal with missing data, and then apply the CPI to the data sets with the imputed observations. Moreover, applying multiple imputations has been found to be a good alternative for the surrogate split strategy, sometimes even leading to a better prediction accuracy [40].

As in any simulation study, only a limited set of conditions was included. Future research could investigate the behavior of the CPI for different data generating processes, with (a) different variable types for the outcome (cf. classification), (b) different variable types for the predictors, (c) other sample sizes, (d) higher numbers of predictors, and (e) different dependency structures between the predictors.

Previous research [5, 34] has shown that the values of both the (unconditional) PI and the CPI are affected by the mtry-value in the tree-growing algorithm. However, the mtry-value also affects the prediction accuracy of the RF. We argue that the mtry-value that optimizes the prediction accuracy of the RF should be used (cf. the simulation study in the Methods section), so that the used importance measures quantify the contributions of the predictors in the optimal RF. Otherwise one risks quantifying and ranking the relevance of the predictors based on a RF that does not predict accurately (or as accurately as possible). Likewise, other hyper parameters in the RF algorithm should preferably also be optimized with respect to prediction accuracy [35], before computing the (C)PI. Future research should investigate the impact of optimizing other hyper parameters on the (C)PI computation and on RF-based variable importance measures in general.

In light of optimizing prediction accuracy, the ntree parameter is not a tuning parameter in a classical sense. Yet it should be sufficiently high to obtain a stable prediction accuracy [29, 35, 41–43]. Moreover, for specific prediction accuracy measures such as the mean squared error (in case of regression) or the Brier score (in case of classification), Probst and Boulesteix [42] have theoretically proven that more trees are always better. However, generally more trees are required for stable variable importance estimates than for prediction purposes [35, 44, 45]. Hence, a sufficiently high ntree-value with respect to prediction accuracy may not suffice for stable (C)PI values. To assess the stability, Strobl and colleagues [29] proposed to fit several RFs with a fixed ntree-value using different random seeds and check whether the rankings of the variables by importance are different between the forests.

Although not observed in the simulation study, the CPI algorithm suggests that under certain conditions the fully conditional permutation scheme (*s*=0) may be too fragmented and thereby lead to meaningless CPI values. Preliminary analyses suggest this may happen when trees are fully grown (combined with large IB/OOB ratios). However, both fully grown trees and using a threshold value of *s*=0 are not recommended in practice. First, literature suggests that fully grown trees do not necessarily lead to optimal prediction accuracy in RFs [35, 46]. Moreover, for larger sample sizes, preventing the trees from fully growing reduces the computing time without substantial loss in prediction accuracy [35, 47]. Second, threshold values of *s*<.5 have limited practical value, because they make the selection of the predictors to condition on too greedy (cf. above). In addition, higher threshold values make this selection more cautious, and thereby automatically reduce the fragmentation of the permutation scheme. Therefore, we believe that — like in our simulation study — overly fragmented permutation schemes are not a practical problem, even when trees are grown deep. Nevertheless, future research should investigate whether and under which conditions the issue of too fragmented permutation schemes could be present.

As an alternative strategy to limit the split points in the permutation scheme, rather than limiting the depth of the trees in the tree-growing algorithm, the depth up to which split points are utilized in the CPI algorithm could be controlled. That is, only split points up to a certain tree depth could be considered for the permutation scheme, a strategy that was suggested by one of the reviewers. Future research could also investigate the potential of this strategy.

In this manuscript we focused on the application of the CPI to identify and rank important predictors in a fitted RF in the spirit of interpretable machine learning. Yet, variable importance measures are also applied in variable selection algorithms, where recursively the most important predictors are selected (or the least important predictors are dropped). Especially in cases were the number of predictors is substantially bigger than the number of observations (*p*>>*n*), such as in gene expression [41] or genome-wide association studies (GWAS) [45], variable selection methods are popular. Although in principle the CPI could also be applied in variable selection algorithms, in practice this may not be feasible because the CPI computation is inevitably slower than computing the PI, or other variable importance measures such as the MDI^14^.

Despite the success of multiple variable importance measures, there is no consensus about the exact meaning of “variable importance”. Although a clear answer may exist for the simple and elementary case where the outcome is a linear combination of independent predictors, in more complex cases (e.g., interaction effects, dependent predictors, non-linear effects, etc.) defining variable importance is far from straightforward. We have made a distinction between a more partial and a more marginal perspective, arguing that the most relevant perspective depends on the research questions at hand. Therefore, when researchers are choosing a variable importance measure, we recommend that they consider which importance perspective corresponds to their research questions.

Finally, the permimp implementation of the CPI is currently applicable to RFs grown using the party or the randomForest R-package. However, in principle, the functionality of the permimp R-package can be extended to RFs fit using other packages as long as (a) the information about the split points and OOB values are available per tree in the RF object, and (b) prediction based on the OOB values is possible per tree.

## Conclusion

In this article we reconsidered the Conditional Permutation Importance [5]. We proposed a new implementation (with an accompanying R-package: permimp) that is generally faster and more stable than the current party implementation. In addition, the new permimp implementation is in accordance with the ensemble-method rationale that characterizes RFs. At the same time, it stays loyal to the original purpose and idea behind the CPI [5].

Using simulated data we illustrated that the issues we identified with the CPI in the party implementation (cf. above) are mitigated in the permimp implementation. From a practical viewpoint the permimp implementation is also more widely applicable, as it is not limited to RFs that are grown using the cforest-function from the party package, but also applies to RFs grown using the randomForest package.

We introduced the threshold-value in the CPI algorithm as a parameter that determines how partial or marginal the CPI is. Depending on the research question, a more partial or more marginal perspective on variable importance may be more adequate.

Finally, the practical relevance of a more partial perspective and the advantage of the new CPI implementation is illustrated in the recent publication of Bierbauer and colleagues [49], in which the CPI was used to identify which variables contribute to the prediction of improvements in exercise capacity of older adults during cardiac rehabilitation.

## Methods

A simulation study was set up to (a) investigate and illustrate the impact of the threshold value *s* on the CPI values, and (b) compare CPI-results according to the party implementation with those from the permimp implementation^15^. Data were generated using three types of data generating processes (DGPs). Hence, for ease of reading, the description of the simulation study is split into three parts. In part 1, we replicated and extended the simulation study of Strobl and colleagues [5], which only includes linear dependencies in the DGP. Because we noticed that this DGP always lead to a very high signal-to-noise ratio (cf. explained variance), we developed an alternative DGP with only linear dependencies in part 2 that always has a 50/50 signal-to-noise ratio. Finally part 3 applied a DGP that also included non-linear (i.e., quadratic) dependencies between the predictors.

***Part 1 - DGP based on the simulation from Strobl and colleagues ***[5]

Within the first DGP, data sets were generated according to a linear regression model with twelve continuous predictors: *Y*_*i*_=***βX***_*i*_+*ε*_*i*_. The twelve predictors ***X*** were sampled form a multivariate normal distribution ***X***∼*N*(***0***,***Σ***), with four conditions for ***Σ***. In each condition, all predictors had unit variance $\left (\sigma _{k,k} = 1, \text {for all} k = 1, \dots, 12\right)$. The four correlation structures ***Σ*** were based on the correlation structure described by Strobl and colleagues [5]: the first four predictors were block-correlated with either *ρ*_*k*,*l*_=0,.1,.5 or.9 for *k*≠*l*≤4, and the other predictors were independent. When *ρ*_*k*,*l*_=0, all predictors were independent, and when *ρ*_*k*,*l*_=.9 this corresponds to the correlation structure applied by Strobl and colleagues.

Like in the simulation study of Strobl and colleagues [5], only six of the predictors were influential. The used regression weights are given in Table 1. The error term was sampled from a normal distribution: *ε*_*i*_∼*N*(0,.5), which lead to a very high signal-to-noise ratio in all conditions.

**Table 1:** Regression Coefficients in the Data Generating Process With Only Linear Dependencies

Simulation	*X*_*k*_	*X*_1_	*X*_2_	*X*_3_	*X*_4_	*X*_5_	*X*_6_	*X*_7_	*X*_8_	*X*_9_	*X*_10_	*X*_11_	*X*_12_
Part 1	*β*_*k*_	5	5	2	0	−5	−5	−2	0	0	0	0	0
Part 2	*β*_*k*_	.1	0	.1	0	.1	0	.1	0	.1	0	.1	0

***Part 2 - alternative DGP with linear dependencies***

In addition to a lower signal-to-noise ratio, we also wanted to reduce the impact of differently sized regression coefficients on the (C)PI-patterns in part 2. Therefore, a DGP was used in which there are only two possible values for the regression weights (see Table 1). In addition, different correlation structures between the predictors were implemented. Within the second DGP, data sets were generated according to a linear regression model with twelve continuous predictors: *Y*_*i*_=***βX***_*i*_+*ε*_*i*_. The twelve predictors ***X*** were sampled form a multivariate normal distribution ***X***∼*N*(***0***,***Σ***), with three conditions for ***Σ***. In each condition, all predictors had unit variance (*σ*_*k*,*k*_=1, for all $k = 1, \dots, 12$). Inspired by the simulation design applied by Grömping [34], correlations between the predictors were set to $\sigma \left (X_{k}, X_{l}\right) = \rho ^{\left (1 + \left \lceil \frac {k}{2}\right \rceil - \left \lceil \frac {l}{2}\right \rceil \right)}$ for *k*<*l*<9 and to zero for 8<*k*<*l*, where ⌈ ⌉ is the ceiling operator. Three values for *ρ* were selected: *ρ*=0,.5, and.9, resulting in three correlation structures. In each correlation structure, the predictors were divided into pairs. Each pair had exactly the same correlation structure with the other predictors.

Like in Part 1, only six of the predictors were influential. The used standardized regression coefficients are given in Table 1. Note that within each pair of predictors based on the correlation structure, one predictor had a regression coefficient equal to 0, while the other predictor had a regression coefficient equal to.1. The error term was sampled from a normal distribution with mean zero, and a standard deviation chosen so that the theoretical explained variance was equal to *R*=.5, which corresponds with a signal-to-noise ratio of one.

***Part 3 - DGP with non-linear dependencies***

The third DGP included ten continuous predictors ***X*** with a non-linear dependency structure. Values for the ten predictors were sampled as follows. Predictors *X*_1_ and *X*_3_ followed a mixture of a standard normal and a uniform distribution:

7 $$ X_{k} \sim \left\{\begin{array}{ll} N(0,1) \quad & \text{if} \,\, Z_{k} = 0\\ U(-2,2) \quad & \text{if} \,\, Z_{k} = 1 \end{array}\right. \quad \text{with} \,\, Z_{k} \sim B(1,.5) \text{, for} \,\, k \in \{1, 3\}.  $$

Predictors *X*_2_ and *X*_4_ were equal to the square of *X*_1_ and *X*_3_, respectively, with additional uniform noise:

8 $$ X_{k} \sim X_{k-1}^{2} + U(-.1,.1) \quad \text{for} \,\, k \in \{2, 4\}.  $$

*X*_5_ and *X*_6_ were bivariate normally distributed with both means $\mu _{X_{5}} = \mu _{X_{6}} = 0$, standard deviations $\sigma _{X_{5}} = \sigma _{X_{6}} = 1$, and a high correlation $\sigma _{X_{5}, X_{6}} =.9$. Finally, predictors *X*_7_ to *X*_10_ were independently standard normally distributed.

There were three conditions (A, B, and C), and in each condition the outcome *Y* was generated according to a different regression model, as presented in Table 2. In all conditions there was both a linear as well as a quadratic effect of *X*_1_ on *Y* and the error term was normally distributed: *ε*∼*N*(0,.5). Condition A only included the linear and quadratic effects of *X*_1_. Condition B had an additional effect of *X*_3_, while condition C had an additional effect of *X*_5_.

**Table 2:** Regression Models in the Data Generating Process With Non-Linear Dependencies Between The Predictors

Option	Formula
Option A	$Y_{i} = X_{1i} + X_{1i}^{2} + \varepsilon _{i}$
Option B	$Y_{i} = X_{1i} + X_{1i}^{2} + X_{3i} + \varepsilon _{i}$
Option C	$Y_{i} = X_{1i} + X_{1i}^{2} + X_{5i} + \varepsilon _{i}$

Because of the design (i.e., the quadratic effect of *X*_1_ on *Y* combined with the quadratic dependence between *X*_2_ and *X*_1_), it can be expected that both *X*_1_ and *X*_2_ have a (relatively) high PI value. In addition, since *X*_1_ and *X*_2_ are linearly independent, we expected that the party implementation would not include *X*_1_ in $Z_{(-X_{2})}^{(s)}$ and (vice versa), so that the CPI patterns for higher threshold values would generally be similar to the unconditional PI patterns. In contrast, the permimp implementation, which is also sensitive to non-linear dependencies, should generally include *X*_1_ in $Z_{(-X_{2})}^{(t)(s)}$ (and vice versa), and therefore lead to a different CPI patterns compared to the PI. To be more precise, we expected that using the permimp implementation, the CPI value for *X*_2_ would be relatively lower compared to the unconditional PI pattern, even for higher threshold values. Similar observations were expected for *X*_4_ in condition B and for *X*_6_ in condition C.

To sum up, there were three types of DGPs — two with only linear dependencies (i.e., part 1 and part 2), and one with non-linear dependencies between the predictors (i.e., part 3) — each with three or four conditions for the data generating process. Combined with a number of observations that could be either *n*=200 or *n*=1000, there were 2×(4+3+3)=20 conditions in total.

***Further simulation specifications***

In each condition 1000 data sets were generated, and each data set was used to fit a RF using the cforest-function in the party R-package, which applies the conditional inference framework for tree-growing [26, 50].

Previous research has indicated that the number of predictors that are randomly pre-selected to be considered for the next split in the tree-growing algorithm (i.e., mtry), has an impact on the pattern of PI and CPI values [5, 34]). However, because we believe that the PI and CPI should be interpreted as methods to quantify the contribution of the predictors to the prediction in a fitted RF, we argue that the mtry-value should be chosen so that the (OOB- or cross-validated) prediction accuracy is optimized. Therefore, for each of the 16 conditions, we compared the prediction accuracy on test data for all possible mtry-values, over 1000 replications^16^. The mtry-value that lead to the average best prediction accuracy was considered as the optimal mtry-value for that condition. This optimal mtry-value was applied to fit the RFs in the simulation study.

In summary, the following specifications were used: ntree =1000 and mtry =*optimal*mtry. In the tree-growing algorithm, when there were (a) less than 20 observations in a node (cf. minsplit = 20), or (b) an new node would have less than seven observations (cf. minbucket = 7) further splitting was prevented. The latter specifications are suggested for fitting unbiased RFs in the party-package [38].

To investigate the impact of the threshold value *s* on the CPI pattern, 16 different threshold values were applied to both implementations^17^. Hence, based on the fitted RF for each data set (a) 16 CPIs according to the party, (b) 16 CPIs according to the permimp implementation, as well as (c) the original PI were computed. When analyzing the results, we mainly focused on the pattern of average CPI values, averaged over the replications. In addition, we also inspected the variability of the CPI values across the replications, by means of the inter-quartile range.

### Results

All the results can be browsed through using a shiny app^18^. In addition to the results, visual representations of the correlation structures applied in part 1 and 2, as well as a scatter plot visualizing the non-linear association between two predictors in part 3 can also be found in the shiny app.

#### Part 1 - DGP based on the simulation from Strobl and colleagues [5]

Because there should be no difference between a more partial and a more marginal perspective on variable importance when all predictors are independent, we expected no impact of the threshold value *s* in the condition where all predictors were uncorrelated (*ρ*=0). In contrast, differing CPI patterns depending on the threshold value were expected when *ρ*>0. We expected bigger pattern changes for higher *ρ*-values. In addition, we did not expect big differences between the party and the permimp implementations because the DGP only included linear dependencies.

##### Impact of the threshold value.

As expected, in the condition without dependencies between the predictors (*ρ*=0), the threshold value *s* does not affect the CPI pattern. At least not relatively: the raw CPI values decrease for lower threshold values, but the relative differences between CPI values (i.e., the pattern of CPI values) stay unchanged. The decreasing CPI values can be explained by the conditional permutation scheme becoming more elaborate and fragmented as the threshold value decreases. In very fragmented permutation schemes, each observation can only be permuted with a limited set of other observations, thereby reducing the potential prediction accuracy reduction caused by permuting the values of a predictor *X*_*k*_. Because there are no dependencies between the predictors, the CPI values for all predictors are equally affected by this mechanism.

Meaningless CPI values are not observed, even when *s*=0. This could imply that the hyper parameters in the tree-growing algorithm minsplit = 20 and minbucket = 7 prevent the permutation scheme form becoming too fragmented. However, especially when *n*=1000 the raw CPI values decrease drastically when *s*≤.5, while for threshold values.8≤*s*<1, the raw CPI values are in the same range of the unconditional PI values. This illustrates that threshold values *s*≤.5 lead to a too greedy selection of the other predictors to condition on.

In the conditions with predictor dependencies (*ρ*>0), when the threshold decreases from *s*=1 to *s*=0 (i.e., when the CPI becomes more conditional), in addition to the above described decrease in raw CPI values, also changes in the relative CPI pattern are observed. This implies that the CPI (with a threshold *s*<1) can lead to different patterns and interpretations than the PI, which is in line with the well known finding that correlated predictors in linear regression lead to different patterns for more partial and more marginal importance measures. And, as expected, higher *ρ*-values lead to bigger pattern changes.

For both implementations, the most relevant CPI pattern changes take place when 0.80≤*s*≤1. Decreasing the threshold value even more only affects the raw CPI values, without relevant changes in the CPI pattern. Only when *ρ*=.1 and *n*=1000 small changes were observed when the threshold value was.5≤*s*≤.8. Furthermore, in the condition where *ρ*=.9 only the CPI values of the correlated predictors change when decreasing the threshold from *s*=1 to *s*=0.95, while the CPI values of the uncorrelated predictors stay constant. These observations contributed to our decision to set *s*=0.95 as the default threshold value in the permimp-package.

##### party vs. permimp implementation.

As can be expected, when the threshold *s*=0 the party and the permimp implementation of the CPI lead to the same results^19^ in all conditions. The most conditional permutation scheme is, by definition, the same in both implementations. Likewise, when the threshold *s*=1 the results of the two implementations are equal, as well as equal to the original PI (cf. above).

In the condition where *ρ*=0, there are no practical differences between the two implementations, regardless of the threshold value. Also in the conditions with correlated predictors (*ρ*>0), the two implementations demonstrate very similar patterns. Only when *ρ*=.1 and the sample size is bigger (*n*=1000), and when *ρ*=.5 and the sample size is smaller (*n*=200) the transition from more to less conditional is slower for the permimp implementation than for the party implementation. This slower transition could be explained by the fact that the permimp implementation selects the other predictors to condition on for every tree separately, so that decreasing the threshold values corresponds with increasing the number of trees for which *X*_*l*_ is included in $Z_{(-k)}^{(t)(s)}$.

A second difference between the two implementations does not relate to the average CPI values, but to the variability in CPI values across the replications. Regardless of the dependency structure between the predictors, when *n*=1000 and 0.1≤*s*≤0.8, the party CPI values show considerably more variability than the permimp CPI values. The sampling noise across the replications seems to substantially affect the party CPI computation, leading to more unstable and hence, less reliable CPI values and rankings. Although generally an increased sample size leads to more stable data analytic results, the opposite is observed for the party CPI implementation. A possible explanation for this counter-intuitive result is given in the “Instability” section above. The permimp implementation does not demonstrate this instability issue.

##### Other observations.

There are two additional observations that do not relate to (a) the used threshold value or (b) the difference between the CPI implementations, but that are relevant for the CPI and PI in general. First, although the same regression weights are used in the different conditions, the CPI patterns consistently differ across the different *ρ*-values, for all applied thresholds. This indicates that the dependence between the predictors does not only affect the PI (cf. [5, 24]), but also the CPI. Even when the CPI is as conditional as possible, the patterns still differ across the four predictor dependency structures. This illustrates that the CPI should be interpreted as a quantification of the contributions of the predictors in a fitted forest and indicates that it does not directly correspond to a parameter in the DGP, even when the DGP is a linear additive model.

Second, the sample size affects not only the raw CPI values, but also the CPI pattern. For instance, in the conditions with *ρ*=.5 or *ρ*=.9 when *n*=200 the CPI values of the two first (correlated) predictors are still higher than the CPI values of the fifth and sixth (uncorrelated) predictors despite their equal absolute regression weights. When *n*=1000, however, the opposite is observed: higher CPI values for the uncorrelated than for the correlated predictors with equal absolute regression weights. This indicates that the finding that the CPI still leads to higher variable importances for correlated predictors [5] cannot be generalized, since it is not observed for bigger sample sizes.

Further research should investigate the cause of the sample size impact. One possibility may be an interaction between the sample size and the tree-growing specifications that prevent further splitting. It could be that for the smaller sample size (*n*=200) the applied (default) specifications (i.e., minbucket = 7 and minsplit = 20) were too restrictive, resulting in an insufficient tree-depth for obtaining optimal prediction accuracy. However, as noted in the discussion section, when tuning hyper parameters, the first aim should be to find the optimal prediction accuracy.

#### Part 2 - alternative DGP with linear dependencies

In general, the obtained results in part 2 were very similar to the results in part 1. Therefore, we focus only on the results that extend the results obtained in part 1.

##### Impact of the threshold value.

Like in part 1, when there are no dependencies between the predictors (i.e., *ρ*=0), the threshold does not affect the (C)PI pattern (relatively). However, when most predictors are correlated (i.e., *ρ*=.5 or *ρ*=.9), decreasing the threshold from *s*=1 to *s*=0 changes the (relative) CPI pattern, with the most relevant CPI pattern changes taking place when 0.90≤*s*≤1. In addition, for threshold values *s*>.9 the CPI values of the uncorrelated predictors don’t demonstrate any changes, but for lower threshold values their raw CPI also start to decrease.

When *ρ*=.5 and *n*=1000 the CPI pattern suddenly jumps when decreasing the threshold value form *s*=.1 to *s*=0. In addition, in the most conditional permutation scheme (*s*=0) the CPI pattern does not correspond to what we would expect from a more partial importance measure. Closer inspection revealed that in this specific condition the partitions in the conditional permutation scheme typically contain only one OOB observation, which makes conditionally permuting the OOB values impossible. Thus, the permutation scheme in the CPI algorithm is too fragmented, which makes the CPI values small and random, and the CPI pattern meaningless. However, in other conditions and for other threshold values (*s*>0) no indications for too fragmented permutation schemes are observed.

##### party vs. permimp implementation.

When the sample size is *n*=1000, the instability issues with respect to the party implementation are observed again. When *ρ*=0 or *ρ*=.9 both implementations lead to very similar average results over the replications. However, when *ρ*=.5, considerable differences between the average CPI patterns of the two implementations are observed when *n*=1000. The CPI computed according to the permimp implementation displays a gradual transition from completely unconditional to as conditional as possible. The CPI according to the party implementation, however, changes drastically when the threshold decreases from *s*=1 to *s*=.995. But then it stays practically unchanged for all 0≤*s*≤0.995.

The fact that in this condition the CPI according to the party implementation is similar to the most conditional CPI as soon as *s*<1 is explained as follows. First, when *ρ*=.5 some of the theoretical pairwise correlations in the correlation structure ***Σ*** are small to very small (i.e.,.125 and.0625). However, for a sample size of *n*=1000 and *s*=0.995 the correlation corresponding to the critical value for the test statistic in the conditional inference framework [33] is equal to $\lvert \sigma _{k,l,0.995}\lvert = \sqrt {\frac {\chi ^{2}_{.995}}{n - 1}} = 0.089$. Therefore, the probability that the pairwise correlations in a specific replication are larger than this critical value, and hence the probability that many other predictors $Z_{(-k)}^{(t)}$ are included in $Z_{(-k)}^{(t)(s)}$ for the first eight predictors *X*_*k*_ is very high, even for *s*=0.995, which can be considered a higher threshold value. We consider this to be a disadvantageous side effect of the party implementation. Especially for higher sample sizes the selection of the predictors to condition on becomes too greedy too fast, so that even practically independent predictors have a high probability of being included in the permutation scheme. The CPI in the permimp implementation mitigates this issue (cf. Fig. 5).

##### Other observations.

Like in part 1, there are differences between the PI and CPI patterns between the conditions where *n*=200 and the conditions where *n*=1000. When *n*=1000 the PI patterns and CPI patterns correspond to what we would expect from a more marginal and a more partial perspective on variable importance, respectively, with clear differences when *ρ*≠0. When *n*=200, however, these differences are less pronounced, which may be related to the signal-to-noise ratio, the sample size, and the applied stopping criteria in the tree-growing algorithm.

#### Part 3 - DGP with non-linear dependencies

Under the DGP with non-linear dependencies between the predictors, we expected that in all conditions the predictor *X*_2_ would have a high PI value due to the combination of its strong quadratic dependence with *X*_1_ and the quadratic effect of *X*_1_ in the DGP. Ideally, we argue, the CPI of *X*_2_ should drop considerably as soon as *s*<1. Similarly, in condition B and condition C, we expected the PI of respectively *X*_4_ and *X*_6_ to be non-zero due to its strong dependence with respectively *X*_3_ and *X*_5_. Ideally the CPI of *X*_4_ would drop to zero as soon as *s*<1. However, we expected that for *X*_4_ in condition B only the CPI in the permimp implementation would display this behavior. The CPI according in the party implementation was expected to require lower threshold values for a similar behavior, because it is only sensitive to linear dependencies between continuous predictors. The drop for the CPI of *X*_6_ in condition C, however, is expected to be observed in both implementations.

##### Impact of the threshold value.

As expected, the PI of *X*_2_ is high in all conditions A, B, and C, despite the fact that its regression coefficient in the DGP is zero. Its PI is even higher than the PI of *X*_1_. Decreasing the threshold form *s*=1 to *s*=0 changes the pattern of CPI values: Generally the CPI of *X*_2_ drops faster and lower than the CPI of *X*_1_. However, there are big differences between the two implementations (see below).

Similar to the results obtained for the DGP with only linear dependencies, from a certain threshold value (*s*=.5) no more relative pattern changes occur, while the raw CPI values keep decreasing. Finally, in condition C the CPI of *X*_5_ and *X*_6_ demonstrate similar behavior as was observed in the condition with only linear dependencies.

##### party vs. permimp implementation.

Due to the very strong dependence between *X*_1_ and *X*_2_ there is an immediate change in the CPI pattern according to the permimp implementation, as soon as *s*<1. For *X*_2_ the CPI decreases considerably (but does not become zero) while the CPI of *X*_1_ demonstrates only a minor change. The party implementation, in contrast, displays a more gradual pattern change, indicating that *X*_1_ is not included in $Z_{(-X_{2})}^{(s)}$, despite the very strong dependence between *X*_1_ and *X*_2_. Similarly, the CPI of *X*_4_ in condition B drops to zero more quickly when decreasing the threshold in the permimp implementation, compared to the party implementation, especially when *n*=1000. Note that given the DGP, we believe that permimp behavior should be preferred over the party behavior.

Similar to the results based on the DGP with only linear dependencies between the predictors, the two implementations lead to the same results when *s*=0 or *s*=1, and when *s*=1 the CPI corresponds with the PI. Under the DGP with non-linear dependencies between the predictors, however, the instability demonstrated by the party implementation is not limited to the bigger sample size conditions (*n*=1000). To some extent it is also observed when *n*=200. In contrast, the permimp implementation does not display any instability. Moreover, the variability of the results across replications becomes smaller with sample size.

## Data Availability

The permimp package is available via https://CRAN.R-project.org/package=permimp. The source code as well as the development version of the permimp package are available via https://github.com/ddebeer/permimp. The full source code for the simulation study as well as the source code for making the figures is available upon request from the corresponding author.

## Abbreviations

RF: Random forest
MDI: Mean decrease in impurity
PI: Permutation importance
CPI: Conditional permutation importance
SNP: Single-nucleotide polymorphism
OOB: Out-of-bag
IB: In-bag
GWAS: Genome-wide association studies
DGP: Data generating process

## Footnotes

1 Predictors or predictor variables are also commonly referred to as features, explanatory or independent variables, and the outcome is also often referred to as dependent variable, criterion or response. Throughout this paper we will consistently use the terms predictor(s) and outcome.

2 In a similar spirit, from Breiman’s 2002 Wald lecture ([21], p.12-14) it is evident that during their work on the original RF algorithm Breiman and Cutler already suggested that, in order to better understand the impact of dependencies among predictors, the predictors should be examined both individually and in combination, as well as in careful conjunction with the respective prediction accuracy [21, 22].

3 This view is in line with the notion of Cutler [22], that variable importance is “an attribute of the fitted forest, not the data (except in so far as the forest was fit to the data)”.

4 We use “party implementation” to refer to the current implementation in the party package (version ≥1.2-4), in which we have already resolved the reported computational issues [6, 24].

5 Small negative differences are possible. This happens when the prediction accuracy after permutation is higher by chance.

6 party::varimp(RFobject, conditional = TRUE)

7 Note that the term “conditional” in the conditional inference framework of Hothorn and colleagues [33] and in the context of the CPI are not related and refer to completely different things. Unfortunately, this double use of “conditional” seems to have led to some confusion among readers and package users. In the conditional inference framework, which is used for unbiased variable selection in the tree and forest algorithms implemented in the party package, the term “conditional” refers to statistical inferences drawn by means of permutation conditionally on the given data set (rather than based on a model for the population). In contrast, the term “conditional” in the context of the CPI refers to permutation conditionally on a grid formed by the other predictors in the RF. In short, the CPI can be computed both for RFs grown using the conditional inference framework, as for RFs grown using other tree-growing algorithms.

8 Or, in the words of Breiman in his 2002 Wald lecture: “The better the model fits the data, the more sound the inferences about the black box are.” ([21], p. 4).

9 Or any other non-linear association that corresponds to a zero correlation.

10 Note that the restriction to linear associations (for continuous variables) also applies to the tree-growing algorithm of the conditional inference trees [26]. However, in practice this is not problematic because of the recursive nature of the tree-growing algorithm. That is, non-linear associations can be picked up and approximated by multiple binary split points.

11 For RFs grown according to the impurity reduction principle, we expect to see the same results that hold for the unconditional PI, namely that the CPI is unbiased with respect to the average importance, but that the sampling variability is higher for variables with more possible split points when bootstrap sampling is used to fit the random forest [38]. Note that for RFs fit using an unbiased tree-growing algorithm like the one proposed by Hothorn and colleagues [26] and when sub sampling rather than bootstrap sampling is used, this issue is avoided.

12 Although we focus on the permimp implementation of the CPI, a similar rationale can be followed pertaining to the party implementation.

13 https://simulations-and-statistics.shinyapps.io/CPI-revisited/

14 For RFs grown according to the original impurity reduction principle [1, 2, 25], there is a variable selection bias in favor of variables with many distinct values or categories that caries over to MDI values [38]. De-biased variants were recently suggested [30, 48]. In addition, for RFs grown using an unbiased tree-growing algorithm like the one proposed by Hothorn and colleagues [26], there is no such bias.

15 In contrast to the party CPI implementation, the permimp implementation is not limited to RFs that are fit using the party R-package. The permimp implementation can also be applied to the original impurity-reduction based RF [1], as implemented in the randomForest R-package [25]. Yet because a comparison of the party and permimp implementations was the main aim of the simulation study, only RFs fit using the party-package were applied.

16 In practice, when the exact data generating process is unknown, cross-validation can be used to find the optimal mtry value.

17 The 16 values were: *s*∈{0,0.1,0.2,0.3,0.4,0.5,0.6,0.7,0.8,0.9,0.925,0.95,0.975,0.99,0.995,1}.

18 https://simulations-and-statistics.shinyapps.io/CPI-revisited/

19 We only observe some random noise, caused by the random permutation.
